# Defining the habenula in human neuroimaging studies

**DOI:** 10.1016/j.neuroimage.2012.08.076

**Published:** 2013-01-01

**Authors:** Rebecca P. Lawson, Wayne C. Drevets, Jonathan P. Roiser

**Affiliations:** a UCL Institute of Cognitive Neuroscience, 17 Queen Square, London, UK; b Laureate Institute for Brain Research and Department of Psychiatry, University of Oklahoma College of Medicine, Tulsa, OK, USA

**Keywords:** Habenula, High-resolution MRI, Segmentation, T2-weighted, Pre-processing, Analysis

## Abstract

Recently there has been renewed interest in the habenula; a pair of small, highly evolutionarily conserved epithalamic nuclei adjacent to the medial dorsal (MD) nucleus of the thalamus. The habenula has been implicated in a range of behaviours including sleep, stress and pain, and studies in non-human primates have suggested a potentially important role in reinforcement processing, putatively via its effects on monoaminergic neurotransmission. Over the last decade, an increasing number of neuroimaging studies have reported functional responses in the human habenula using functional magnetic resonance imaging (fMRI). However, standard fMRI analysis approaches face several challenges in isolating signal from this structure because of its relatively small size, around 30 mm^3^ in volume. In this paper we offer a set of guidelines for locating and manually tracing the habenula in humans using high-resolution T1-weighted structural images. We also offer recommendations for appropriate pre-processing and analysis of high-resolution functional magnetic resonance imaging (fMRI) data such that signal from the habenula can be accurately resolved from that in surrounding structures.

## Introduction

The habenula comprises a pair of small nuclei adjacent to the posterior end of the medial dorsal (MD) thalamus and in many vertebrates can be divided into medial (MHb) and lateral (LHb) portions (Andres et al., 1999). The dorsal conduction stream provides largely distinct pathways relayed by the MHb and LHb (Bianco and Wilson, 2009). The MHb primarily receives inputs from the septum and projects to the interpeduncular nucleus (IPN). The globus pallidus internus, lateral septo-hypothalamic continuum and suprachiasmatic nucleus primarily, but not exclusively, provide the subcortical inputs to the LHb (Bianco and Wilson, 2009) whilst its efferent projections include inhibitory neurons in the rostromedial tegmental nucleus (rMTG), which synapse onto dopamine neurons in the ventral tegmental area and the substantia nigra (Jhou et al., 2009). The LHb also has substantial reciprocal anatomical connections with serotonergic neurons in the median and dorsal raphe nuclei (Herkenham, 1979) and in rats receives a direct cortical projection from prelimbic frontal cortex (Beckstead, 1979).

This unique position, as a hub between corticolimbic and midbrain monoaminergic regions, could allow positively or negatively valenced states or stimuli to modulate motor output, consistent with the hypothesis that the habenula plays a critical role in the motivational aspects of reinforcement learning and decision-making, as extensively reviewed elsewhere (Hikosaka et al., 2008; Hikosaka, 2010). The habenula responds to primary aversive stimuli (Matsumoto and Hikosaka, 2009), cues that predict aversive stimuli (Matsumoto and Hikosaka, 2007), and appetitive stimuli that are less rewarding than predicted (Matsumoto and Hikosaka, 2009). Its ability to inhibit dopamine neuron firing, via the rMTG, has been established in rodents (Ji and Shepard, 2007) and non-human primates (Matsumoto and Hikosaka, 2007). Furthermore, evidence from rodent models of depression suggests a role for the habenula in learned helplessness behaviour (Caldecott-Hazard et al., 1988; Shumake and Gonzalez-Lima, 2003), leading to the hypothesis that habenula dysfunction may play an important role in major depression (Morris et al., 1999; Sartorius et al., 2010).

### Studies of the habenula in humans

Advances in high-resolution T1-weighted image acquisition have been exploited in two recent structural MRI studies of the habenula in humans. One study comparing habenula volume between groups of healthy volunteers, unipolar and bipolar depressed populations acquired T1-weighted structural images with a resolution of 550 μm isotropic, and demonstrated that habenula volume is decreased in unmedicated, depressed bipolar patients and unipolar females, relative to controls (Savitz et al., 2011a). Another high-resolution structural study of habenula volume found no significant difference between healthy controls and individuals with post-traumatic stress disorder (Savitz et al., 2011b), consistent with post-mortem data suggesting that altered habenula volume may be relatively specific to depression (Ranft et al., 2010).

A handful of fMRI studies have reported habenula blood oxygen-level-dependent (BOLD) responses to making errors and receiving negative feedback on a range of different tasks (Ide and Li, 2011; Li et al., 2008; Noonan et al., 2011; Ullsperger and von Cramon, 2003) and also recently to noxious heat stimulation (Shelton et al., 2012). These studies used standard fMRI acquisition protocols, with images of slice thickness 3–5 mm and pre-processing steps that included spatial normalisation to the Montreal Neurological Institute (MNI) brain template and the application of smoothing kernels between 5 and 10 mm full-width at half-maximum (FWHM) prior to group-level voxel-wise analysis. One BOLD fMRI study used a different approach (Salas et al., 2010), acquiring higher resolution images (2 mm isotropic) and manually co-registering functional and structural images without applying smoothing. A cubic 6 mm region of interest (ROI) was then placed manually in the vicinity of the left and right habenula for each subject, and for subsequent analysis habenula voxels were defined as those correlating negatively with BOLD signal in the ventral striatum during a separate fMRI time series. Using a fixed-effects analysis this study reported habenula responses to omission of expected reward. Finally, one study used arterial spin labelling to measure habenula perfusion during the resting-state in remitted depressed patients and controls following acute tryptophan depletion (Roiser et al., 2009). This study acquired images of 4 mm slice thickness, outlining the habenula manually for each subject on T1-weighted structural images prior to automated co-registration between structural and functional images following global normalisation.

### Challenges to analysing the habenula in human fMRI

There exist a number of challenges in identifying responses from the human habenula using fMRI. Post-mortem estimates of total (medial plus lateral) habenula volume in humans are ~ 31 mm^3^ on the left and ~ 33 mm^3^ on the right (Ranft et al., 2010) (though these estimates do not account for the expected post-mortem brain shrinkage: Stockmeier et al., 2004). This volume approximates the size of a single image voxel acquired in standard fMRI protocols. Moreover, existing data from structural MRI scans reported combined habenula volumes ~ 30–36 mm^3^ (Savitz et al., 2011a, 2011b) suggesting that in each hemisphere the habenula may be even smaller than the standard functional MRI voxel size.

Voxel-wise approaches to fMRI analysis across subjects typically employ several pre-processing steps to account for inter-individual variability in brain structure, including spatial normalisation to a standard stereotaxic space and substantial smoothing. Although these steps are advantageous for locating signal from larger structures, typically-employed smoothing kernels are larger than the habenula itself, raising the possibility that signal measured over the stereotaxic atlas coordinates for the habenula may include substantial components that emanate instead from adjacent structures, such as the MD thalamus or the epithalamic paraventricular nucleus.

Recent advances in high-resolution fMRI sequence development permit the acquisition of 1.5 mm isotropic *functional* images on 3 T MRI scanners and have permitted the study of fine grained microstructure and small nuclei such as the periaqueductal grey (PAG) (Mobbs et al., 2007). Here we outline a detailed approach to anatomically define the habenula for human high-resolution MRI studies in conjunction with a stereotactic atlas of the human brain (Mai et al., 2008). Our procedure involves manually reorienting each structural image into close alignment with the atlas to permit accurate habenula definition according to clear anatomical landmarks without normalisation. When the tracings had been made, inter-rater reliability correlation coefficients for volume were calculated for left, right and combined habenula volume. We also provide estimates of habenula position and volume in Montreal Neurological Institute (MNI) space, as well as accompanying reliability statistics. Our analyses suggest that even with the best available spatial normalisation (DARTEL), achieving excellent registration between subjects, variation across individuals in post-normalisation habenula location would still necessitate the employment of smoothing kernels of a similar size to that of the habenula itself for voxel-wise group-level analyses, running the risk of contaminating signal ostensibly measured in this structure from surrounding regions.

## Materials and methods

### Participants

Twenty-four healthy adults (15 females, mean age = 26 years, standard deviation (SD) = 4.7, range 20–37) participated in this study. All reported no history of neurological or psychiatric illness and were screened for standard MRI contraindications in advance of testing. Participants were compensated for their time and provided written informed consent, as approved by the London Queen Square Research Ethics Committee.

### Imaging parameters

High-resolution T1-weighted anatomical images were acquired with a Siemens Magnetom Trio 3 T MRI scanner using a 32-channel phased-array head coil and an optimised 3D MDEFT imaging sequence with correction for B1 inhomogeneities at 3 T (Deichmann, 2006). Image resolution was 770 μm isotropic (matrix size = 304 × 288 × 224, TR = 7.92 ms, TE = 2.48 ms, excitation flip angle = 16°). A single 17-minute scan was acquired for each participant.

### Manual reorienting

Anatomical images were viewed and reoriented using Statistical Parametric Mapping (SPM8, Wellcome Trust Centre for Neuroimaging, London, http://www.fil.ion.ucl.ac.uk/spm/software/spm8/). Each subject's anatomical image was manually reoriented such that the coordinates [x = 0, y = 0, z = 0] occupied the midpoint of the anterior commissure (AC) with deviations from the origin now defined relative to the AC, the image was rotated and translated as necessary until the x and z co-ordinates of the midpoint of the posterior commissure (PC) were the same as for the AC (i.e. x = 0, z = 0); in other words the brain was oriented along the AC–PC line passing through the centres of each commissure, after Mai et al., 2008). Finally, the image was viewed in coronal section and rotated as necessary to ensure symmetry across a sagittal plane that included the AC–PC line. With the anatomical images now aligned (but not spatially transformed) to the same reference frame as our stereotactic atlas (Mai et al., 2008), the habenula was defined using anatomical landmarks. We note that MNI space is oriented slightly differently to the Mai atlas (the z co-ordinate of the PC is approximately − 4.5 in MNI space) and as such the procedure we describe below may not be accurate for images oriented to MNI space.

### Anatomical delineation

**Fig. 1:**
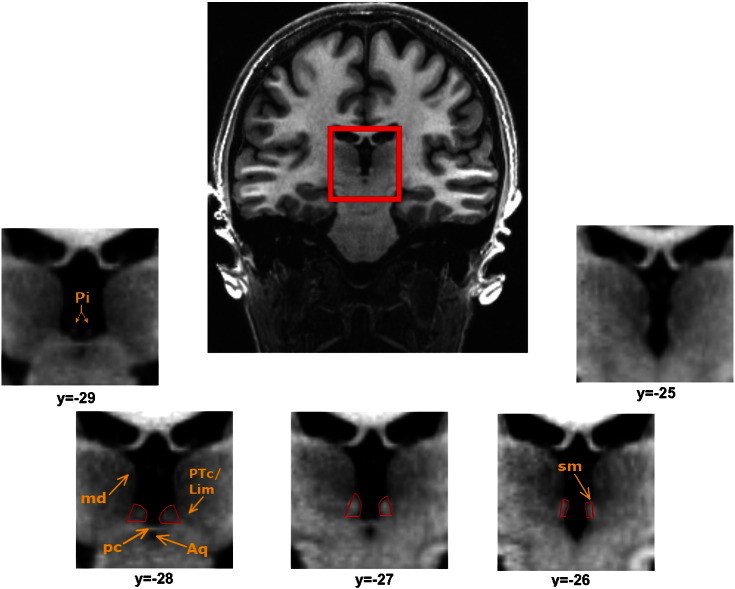
Coronal MRI sections of the habenula (outlined in red) and neighbouring anatomical landmarks on T1-weighted MRI images. The habenula appears brighter than the adjacent mediodorsal thalamic nucleus due to relatively dense white matter fibres. In the more posterior planes the habenula protrudes into the third ventricle and appears pyramidal in shape; moving more anterior, the habenula shifts slightly dorsally and appears more oblong in shape. An individual representative subject is shown. Note that the y co-ordinate of the PC will vary across individuals. Aq = cerebral aqueduct, pc = posterior commissure, Pi = pineal stalk, md = mediodorsal thalamic nucleus, lim/pc = limitans nucleus/pretectal area, sm = stria medullaris.

The habenula lies immediately dorsal to the PC and anterior to the pineal gland, and occupies approximately the same z-coordinate as the latter structure, around 1–5 mm dorsal to the AC–PC line. Moving anterior approximately 1 mm from the most anterior extent of the pineal stalk in coronal sections, the posterior aspect of the habenula can be seen protruding into the third ventricle on either side of the midsaggital plane (Fig. 1). Due to the extensive white matter plexuses contained within the habenula, this structure's density appears brighter than the adjacent thalamic grey matter on T1-weighted images, aiding its delineation from surrounding grey matter and cerebrospinal fluid (CSF).

**Fig. 2:**
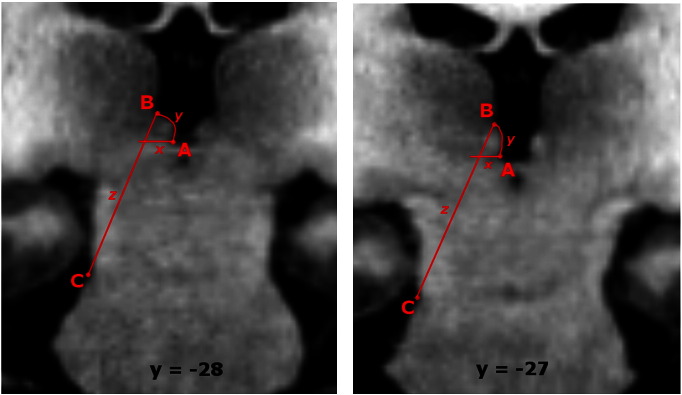
Detail of the geometrically defined protocol for delineating the habenula in posterior slices. See text for explanation of points A, B and C and connecting lines x, y and z.

For each participant the left and right habenula were segmented using MRIcron (http://www.mccauslandcenter.sc.edu/mricro/mricron/) by colouring individual voxels. The following geometric procedure is an extension of the method described in Savitz et al. (2011a, 2011b) although see Supplementary materials for methods and results pertaining to ROIs defined based on image contrast alone. At a slice thickness of 770 μm the habenula spanned a minimum of three and a maximum of five coronal slices from its most posterior to its most anterior aspect (Fig. 1). In posterior slices, defined as those where the PC (or the habenular commissure (HC) if clearly separate from the PC) is visible, the lateral boundary is difficult to delineate from neighbouring dorsolateral structures using image contrast alone. In order to define these boundaries objectively a geometric method was used (Fig. 2). Point A was the intersection between the medial boundary of the habenula and the PC/HC. Next point B was defined as the dorsal point of the medial habenula boundary where the curve of the medial boundary meets the MD. Finally point C was defined as the lateral aspect of the mesopontine jct. next to the tentorial incisure. These three points were easily identifiable in all subjects. In these slices lateral edge of the habenula was defined by a straight line drawn between points ‘B’ and ‘C’ (line ‘z’). The medial extent of the ventral boundary was formed by drawing a straight line extending horizontally and laterally from point A (line ‘z’). The ventrolateral apex of the habenula was defined as the intersection of the lines ‘z’ and ‘x’. The medial boundary was defined by the CSF of the third ventricle in all slices, and connected points A and B in posterior slices (line ‘y’). Ambiguous (dark grey) voxels at the medial boundary were included in the ROI as they likely contain part of the habenula. In the most anterior slice(s), where the PC/HC is not present, the white matter of the stria medullaris of the thalamus and the fasciculus retroflexus provide sufficient image contrast to delineate the dorsal, lateral and ventral edges (Fig. 1). The posterior boundary was defined as the most posterior slice containing the PC/HC in which the habenula was still present (as opposed to CSF or the most anterior extent of the pineal gland). The anterior boundary was identified as the most anterior slice with the absence of bright habenular tissue protruding into the CSF of the third ventricle and also the presence of the dorsal tip of the stria medullaris. The medial and lateral habenular nuclei could not be reliably distinguished from each other and accordingly were combined into a single habenula region.

### Reliability analysis

The habenula was segmented independently by two raters using the protocol described above. Habenula ROIs (binary masks) were imported into the Marsbar (Brett et al., 2002a) toolbox for SPM where volume and centre-of-mass coordinates were obtained. We calculated the Model 2 intraclass correlation coefficient (ICC) (Shrout and Fleiss, 1979) for left, right and combined habenula volume in native space using SPSS 20 (IBM, Chigago, IL). Our ICC analysis was based on single-measures data and sought absolute agreement between raters. ICC coefficients of 0.81–.1.00 are considered ‘almost perfect’, 0.61–0.80 are ‘substantial’, 0.41–0.60 are ‘moderate’, 0.21–0.40 are ‘fair’ and 0.00–0.20 are ‘slight’ (Landis and Koch, 1977). ICCs for normalised left and right habenula coordinates were also calculated.

### Normalisation

Each subject's anatomical image, and the left and right habenula ROIs in register with it, were normalised to MNI space using DARTEL (Ashburner, 2007). This SPM toolbox combines several methodological advances to address the limitations of standard normalisation routines using discreet cosine transform, and provides better normalisation than previous SPM approaches and alternative nonlinear methods for image registration (Klein et al., 2009; Peelle et al., 2009). Normalisation of the T1 images was a multi-stage procedure that first included segmentation into grey and white matter tissue classes which were imported into a format to which the DARTEL algorithm can be applied. The deformations that best aligned our subject images were then estimated and average templates were created. Next spatial normalisation parameters were generated for transforming the grey matter component of our DARTEL template image to the segmented grey matter component of the SPM single subject template image in MNI space. Finally, in order to map from each individual structural scan to DARTEL template to MNI space, the MNI-to-template spatial normalisation parameters were combined with the deformations estimated by DARTEL and applied to the anatomical image and the habenula ROIs for each subject. At each stage and for each image, the acquisition resolution was preserved.

## Results

### Native space

**Fig. 3:**
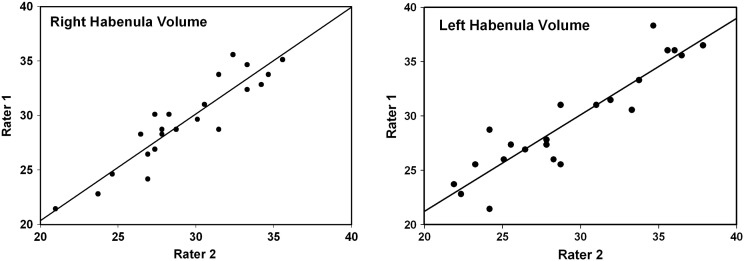
Left and right habenula volumes in native space, as determined by two independent raters.

Mean habenula volume was 29.3 mm^3^ (SD 3.7, range 21.2 to 35.3) on the right and 29.4 mm^3^ (SD 4.7, range 22.6 to 37.2) on the left, in agreement with the volumes measured post-mortem (Ranft et al., 2010). The ICC values for the habenula volumes were in the “almost perfect” range: 0.922 (confidence interval (CI) = 0.828–0.965) for the right and 0.920 (CI = 0.824–0.964) for the left habenula (Fig. 3). Combined habenula volume was 58.7 mm^3^ (SD 6.3, range 50.4-71.4) and the ICC for combined habenula volume was 0.897 (CI = 0.779–0.954).

### Normalised space

The brain size for the MNI template is larger than that represented by the stereotaxic array of Talaraich and Tournoux (Brett et al., 2002b) and the default smoothing applied to these templates (8 mm), whilst helpful to account for inter-subject variability in large cortical structures, will likely inflate the size of smaller structures in the brain. Accordingly, normalisation from native to MNI space, on average, increased habenula volume in most participants. Mean normalised habenula volume was 44.6 mm^3^ (SD 5.8, range 31.7-60.2) on the right and 43.2 mm^3^ (SD 7.7, range 31.0–58.0) on the left. For the normalised right habenula, the mean x-coordinate was 4.8 (SD 0.39, range 4.3 to 6.0), the mean y-coordinate was − 24.1 (SD 0.55, range − 23.4 to − 25.3) and the mean z-coordinate was 2.2 (SD 0.47, range 1.4 to 3.5). For the normalised left habenula, the mean x-coordinate was − 2.8 (SD 0.4, range − 2.0 to − 3.7), mean y-coordinate was − 24.4 (SD 0.59, range − 23.4 to − 25.6) and mean z-coordinate was 2.3 (SD 0.53, range 1.3 to 3.7). The ICC for normalised habenula volume was numerically smaller than for the native space habenula volume; 0.870 (CI = 0.723–0.942) on the right and 0.902 (CI = 0.789–0.956) on the left. The ICC for the normalised right habenula centre of mass was 0.907 (CI = 0.882–0.965) for the x-coordinate, 0.966 (CI = 0.924–0.985) for the y-coordinate and 0.943 (CI = 0.873–0.975) for the z-coordinate. The ICC for normalised left habenula centre of mass was 0.979 (CI = 0.952–0.991) for the x-coordinate, 0.994 (CI = 0.986–0.997) for the y-coordinate and 0.967 (CI = 0.926–0.986) for the z-coordinate.

## Discussion and conclusion

Using high-resolution structural MRI we report a reliable and reproducible protocol for anatomically locating and segmenting the habenula in humans. The level of anatomical detail afforded by high resolution (770 μm) T1-weighted images combined with manual reorienting and the application of a geometric method in posterior slices allowed us to form the boundaries of the habenula in all planes whilst ensuring that the ROI does not include neighbouring structures. The reliability of this protocol, as measured by the ICC, was very high in native space ROIs.

Our analysis raises concerns about the suitability of standard fMRI acquisition, pre-processing and analysis procedures for investigating the function of this small subcortical structure (though we note that these issues also apply to the study of any structure of similarly small size). It is of particular note that the range of the post-normalisation x-, y- and z-coordinates across subjects was approximately 2 mm in each plane, which corresponds to approximately two-thirds of the size of the habenula. This means that, even with high-resolution T1-weighted images, initial manual reorientation to set the AC at the origin and the best available normalisation, the maximum localisation error between two individuals may be greater than the radius of the habenula itself.

With respect to *functional* neuroimaging, assuming one acquires high-resolution (1.5 mm isotropic) fMRI data, a smoothing kernel approximately equal to the size of the habenula itself (~ 2–3 mm) would be necessary to account for this inter-subject variability in location. However, at standard fMRI voxel size (~ 3 mm isotropic) this equates to no smoothing at all. The smoothing kernels applied in past studies reporting habenula responses ( 5-12 mm FWHM) will almost certainly have resulted in contamination of the observed signal from adjacent structures, as acknowledged by some authors (Noonan et al., 2012), indeed even where smoothing has not been applied contamination of signal from neighbouring structures has been noted (Salas et al., 2010). The effect of such contamination is unclear: if signal from structures that do not show qualitatively similar patterns of response to the habenula is included, this could reduce the magnitude of any observed effect and result in false negative results; alternatively, misleading positive results could be observed if signal that truly emanates from regions adjacent to the habenula (e.g. MD thalamus) is included in analysis.

We also note that the reliability of the habenula volume (as assessed by the ICC) is reduced following normalisation (0.870 right and 0.902 left in normalised space versus 0.922 right and 0.920 left in native space) and normalised habenula ICCs had lower bound confidence intervals that were in the ‘moderate’ range. This suggests that normalisation, whilst performing accurately in terms of habenula centre of mass, could introduce errors in habenula volume. As such, volumetric studies of the habenula should consider analysis in native space as an alternative to voxel-wise approaches such as voxel-based morphometry.

Even where high-resolution images have been acquired, there are a number of approaches that researchers can adopt to minimise any reductions in spatial specificity and to avoid introducing localisation errors during pre-processing. High-resolution functional images are particularly susceptible to movement artefacts, so investigators need to check thoroughly that between-scan movement in each subject does not exceed conservative criteria (e.g., ~ one-half a voxel in translation or ~ one-half a degree in rotation). In addition, the output from automated realignment procedures should be visually inspected. Automated co-registration between modalities (i.e., registration of functional to structural scans) will be superior at higher resolutions as increased mutual information in both images can be exploited to great effect by packages employing 3D rigid-body transformations, circumventing the need for subjective manual co-registration. Furthermore, the quality of registration to structural images will be improved by unwarping EPI images with a field-map; especially at higher magnetic field strength (Hutton et al., 2002). However, every pre-processing step that requires re-slicing (or involves inherent smoothing) will lead to a slight degradation the spatial resolution (Woods et al., 1998). Consequently, avoiding a spatial normalisation step (as we propose here) should lead to less localisation error.

We acknowledge that, even with high-resolution T1-weighted images, it can often remain challenging to visualise adequate tissue properties and obtain sufficient contrast to delineate all the boundaries of the habenula, especially the lateral boundary in the most posterior slices. It is for this reason that we have taken a geometric approach in defining this boundary. However, it is possible that alternative pulse sequences or combinations of sequences (Solano-Castiella et al., 2011) could enhance the tissue contrast resolution for the habenula. In addition, advances in anatomical imaging permitting whole brain mapping of T1, T2*, proton density (PD) and magnetization transfer (MT) images, which are sensitive to iron concentration, water content and myelination, conceivably may open up new routes to study subcortical microanatomy in vivo (Helms et al., 2009). These methods remain a possibility for future application to the habenula.

In conclusion, our findings have three important implications. First, accurate definition of the function of the habenula in humans is likely to require high-resolution neuroimaging. Second, using a subject-specific ROI approach habenula analysis is straightforward and reliable, and at the group level will likely produce more robust results than a voxel-wise analysis due to the variability of this structure's location in spatially transformed images. Third, the use of smoothing kernels larger in size than the habenula should be avoided since this will likely contaminate any observed habenula responses with signal from nearby structures. We hope that the procedures laid out in this technical report will be useful to other investigators investigating the structure and function of the human habenula.
