# Supplementary material for: Defining the habenula in human neuroimaging studies
Source: Neuroimage. 2013 Jan 1;64:722–7. doi: 10.1016/j.neuroimage.2012.08.076 (PMC3650642; doi:10.1016/j.neuroimage.2012.08.076)
Supplement: Supplementary file 1 — Supplementary materials. [file mmc1.docx]

**Supplementary Materials**

**Supplementary Methods**

Anatomical delineation using image contrast

With high-resolution T1-weighted images, it is possible to delineate the boundaries of the habenula using anatomical landmarks alone (Savitz et al., 2011) although it can remain challenging to visualise adequate tissue properties and obtain sufficient contrast to do so. While we feel that the ‘geometric’ method reported in the main article is more objective and easily reproducible across labs, the following ‘contrast’ method is presented here for comparison and reference. For each participant the left and right habenula were segmented using the following procedure, drawn in MRIcron (<http://www.mccauslandcenter.sc.edu/mricro/mricron/>) by colouring individual voxels. The medial boundary was defined by the CSF of the third ventricle. Ambiguous (dark gray) voxels at the medial boundary were included in the ROI as they likely contain part of the habenula. The medial extent of the ventral boundary in the most posterior slice was formed by the dorsal edge of the white matter of the PC (or the habenular commissure (HC) if clearly separate from the PC) and sloped ventrolaterally to its most lateral extent, just below the dorsal edge of the PC/HC. The dorsal and lateral boundaries were determined by the mediodorsal thalamic nucleus, limitans nucleus, or pretectal area in posterior slices and by the white matter of the stria medullaris of the thalamus in the most anterior slice. At a slice thickness of 770um the habenula spanned a minimum of three and a maximum of five coronal slices from its most posterior to its most anterior aspect. The posterior boundary was defined as the most posterior slice containing the PC/HC in which the habenula was still present (as opposed to CSF or the most anterior extent of the pineal gland). The anterior boundary was identified as the most anterior slice with the absence of bright habenular tissue protruding into the CSF of the third ventricle and also the presence of the dorsal tip of the stria medullaris. The medial and lateral habenular nuclei could not be reliably distinguished from each other and accordingly were combined into a single habenula region.

**Supplementary Results**

Native space

Mean habenula volume was 32.9mm³ (SD 4.7, range 25.5 to 41.0) on the right and 31.6mm³ (SD 5.0, range 23.3 to 39.2) on the left, in agreement with the volumes measured post-mortem (Ranft et al., 2010). The ICC values for the habenula volumes were in the “almost perfect” range: 0.935 (confidence interval (CI) = 0.867-0.971) for the right and 0.904 (CI = 0.787-0.956) for the left habenula. Combined habenula volume was 64.5mm³ (SD 7.4, range 53.4 to 78.4) and the ICC for combined habenula volume was 0.938 (CI = 0.862-0.973).

Normalised space

Mean normalised habenula volume was 49.9mm³ (SD 5.6, range 38.8-63.5) on the right and 46.7mm³ (SD 8.1, range 31.5-61.2) on the left. For the normalised right habenula, the mean x-coordinate was 4.9 (SD 0.41, range 4.3 to 6.1), the mean y-coordinate was -24.3 (SD 0.58, range -23.4 to -25.5) and the mean z-coordinate was 2.2 (SD 0.49, range 1.1 to 3.2). For the normalised left habenula, the mean x-coordinate was -2.9 (SD 0.4, range -2.2 to -4.1), mean y-coordinate was -24.5 (SD 0.58, range -23.5 to -25.7) and mean z-coordinate was 2.4 (SD 0.53, range 1.4 to 3.4). The ICC for normalised habenula volume was numerically smaller than for the native space habenula volume; 0.829 (CI = 0.648-0.922) on the right and 0.860 (CI = 0.707-0.937) on the left. The ICC for the normalised right habenula centre of mass was 0.959 (CI = 0.909-0.982) for the x-coordinate, 0.979 (CI =0.947-0.991) for the y-coordinate and 0.953 (CI = 0.889-0.980) for the z-coordinate. The ICC for normalised left habenula centre of mass was 0.963 (CI = 0.917-0.984) for the x-coordinate, 0.982 (CI = 0.959-0.992) for the y-coordinate and 0.942 (CI = 0.869-0.975) for the z-coordinate.

Method agreement

Cronbach’s Alpha shows a strong agreement between this supplemental ‘contrast’ method and recommended ‘geometric’ method reported in the main article (0.728 for the right and 0.898 for the left habenula using the average of the volumes across the raters).
